# A temporal trophic shift from primary parasitism to facultative hyperparasitism during interspecific competition between two coevolved scelionid egg parasitoids

**DOI:** 10.1002/ece3.8483

**Published:** 2021-12-20

**Authors:** Tim Haye, Jinping Zhang, Marion Risse, Tara D. Gariepy

**Affiliations:** ^1^ CABI Delemont Switzerland; ^2^ MARA‐CABI Joint Laboratory for Bio‐safety Institute of Plant Protection Chinese Academy of Agricultural Sciences Beijing China; ^3^ London Research and Development Centre Agriculture and Agri‐Food Canada London Ontario Canada

**Keywords:** extrinsic competition, hyperparasitoid, intrinsic competition, multiparasitism, *Trissolcus*, trophic interactions

## Abstract

Understanding competition between scelionid parasitoids that exploit the same host may provide insight into strategies that allow coexistence on a shared resource. Competition studies typically focus on interactions between native and exotic parasitoids that do not share an evolutionary history; however, coevolved parasitoids may be more likely to demonstrate strategies to avoid or exploit a shared resource. We examined intrinsic and extrinsic competition between Asian *Trissolcus japonicus* (Ashmead) and *T. cultratus* (Mayr) (Hymenoptera: Scelionidae) associated with *Halyomorpha halys* (Stål) (Hemiptera: Pentatomidae) that share an evolutionary history. Interspecific interactions were assessed by providing parasitized egg masses to each species at various intervals post‐parasitism, and measuring host acceptance, developmental suitability, and guarding behaviour. *Trissolcus japonicus* showed high acceptance of parasitized hosts up to 72 h following oviposition by *T. cultratus*, despite a very poor developmental outcome. In contrast, *T. cultratus* generally avoided ovipositing in *H. halys* eggs containing *T. japonicus* early‐instar larvae but did not avoid parasitizing *H. halys* that contained eggs and third instar larvae. The adaptive value of this behaviour was supported by developmental outcome: *T. cultratus* outcompeted *T. japonicus* eggs but not early‐instar larvae, and a trophic shift occurred wherein *T. cultratus* developed as a facultative hyperparasitoid on third instar *T. japonicus* larvae. *Trissolcus japonicus* guarded egg masses 8–12× longer and displayed more aggressive interactions than *T. cultratus*, suggesting *T. japonicus* is the superior extrinsic competitor. Development as a facultative hyperparasitoid provided a competitive niche for Asian *T. cultratus* and confirms its instrinsic competitive superiority. This also occurs in a biologically distinct European population of *T. cultratus*, suggesting that facultative hyperparasitism as a competitive strategy is retained in geographically separated populations that have not coevolved with *H. halys* or *T. japonicus*.

## Graphical abstract

Competition between two co‐evolved scelionid egg parasitoids was evaluated to better understand the competitive strategies that allow these two species to coexist on the same host. Not only was *Trissolcus cultratus* the superior intrinsic competitor when it was the first species to parasitize an egg mass, but it was also able to develop as a facultative hyperparasitoid on *T. japonicus*. In contrast, *T. japonicus* displayed more aggressive interactions and longer guarding times, indicating that it is the superior extrinsic competitor.

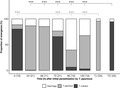

## 1 INTRODUCTION

Parasitoids play a vital role in ecosystems as regulators of herbivorous insects, and interspecific competition can influence the nature and outcome of interactions across different trophic levels (Boivin & Brodeur, 2006). Although the competitive exclusion principle dictates that no two species can coexist in the exact same niche, the reality is that what appears as coexistence can generally be accounted for by adaptation of one or both species to permit co‐occurrence (Hardin, 1960). Parasitoids that share the same host resource are likely to encounter hosts that are already parasitized and will either accept or reject the host depending on whether they can detect the presence of another parasitoid, and whether this makes the host less suitable for development of their own offspring (Harvey et al., 2013). Host suitability in this context depends on the probability of offspring survival from the species that oviposits in an already‐parasitized host (van Baaren et al., 1995). Parasitoids will generally avoid ovipositing in a host that has already been parasitized either by the same species (superparasitism) or a different species (multiparasitism); and avoidance often increases with the time between the first oviposition and a subsequent encounter, but decreases when there is limited availability of unparasitized hosts (Hubbard et al., 1987). However, the ability to discriminate between parasitized and unparasitized hosts is likely more frequent at the intraspecific level versus the interspecific level, due to the fact that parasitoids are more likely to encounter hosts already parasitized by conspecifics (van Alphen & Visser, 1990; Vinson, 1972).

Hymenopteran egg parasitoids in the family Scelionidae have become of considerable interest due to their potential as classical biological control agents of invasive pests, including *Halyomorpha halys* (Stål), *Nezara viridula* L., and *Bagrada hilaris* (Burmeister) (Hemiptera: Pentatomidae) (McPherson, 2018). The recent adventive establishment of Asian *Trissolcus japonicus* (Ashmead) (Hymenoptera: Scelionidae) in North America and Europe has resulted in interest in the impact of this parasitoid on the globally invasive *H. halys*. There are also concerns regarding indirect non‐target effects due to competitive interactions between *T. japonicus* and native egg parasitoids (Gariepy & Talamas, 2019; Konopka et al., 2017). However, interest in competitive interactions among Scelionidae goes beyond an applied biological control context, as the presence or absence of a shared evolutionary history may dictate whether species have developed mechanisms to coexist on a shared resource (Cusumano et al., 2016; Wang et al., 2008).

Asian *T. japonicus* and *T. cultratus* (Mayr) have a shared evolutionary history, are the most common egg parasitoids of *H. halys*, and overlap in time of occurrence and habitat (Avila et al., 2021; Zhang et al., 2017). As such, it is likely that these species will encounter already‐parasitized eggs in the field, and differences in reproductive strategy have likely evolved to allow coexistence on the same host resource. *Trissolcus cultratus* is known to occur in Asia and Europe; however, despite being morphologically identical, biological differences have been observed (Talamas et al., 2019). Of particular interest, is the inability of European *T. cultratus* to develop on *H. halys*, whereas its Asian counterpart successfully exploits *H. halys* (Konopka et al., 2017). Unsuccessful development in *H. halys* has created an evolutionary trap for European *T. cultratus* (Abram et al., 2014); however, it was demonstrated that European *T. cultratus* can develop as a facultative hyperparasitoid on *H. halys* eggs parasitized by *T. japonicus*, thereby providing an escape from the evolutionary trap in an otherwise developmentally unsuitable host (Konopka et al., 2017).

As European *T. cultratus* and Asian *T. japonicus* are geographically separated, the pressure to develop strategies to coexist on the same host are presumably absent, and therefore it raises the question as to whether facultative hyperparasitism is an adaptive phenomenon conserved within *Trissolcus*. In particular, whether this strategy is conserved across geographically separated populations of *T. cultratus*, which have critical differences in their biology. Here we investigate competitive interactions between Asian *T. cultratus* and *T. japonicus* larvae (intrinsic competition) and adults (extrinsic competition) in the native range where they share an evolutionary history in order to shed some light on the mechanisms that allow the two egg parasitoid species to share a resource, and to determine whether facultative hyperparasitism is retained as an adaptive competitive strategy across geographically separated populations of *T. cultratus*.

## 2 MATERIAL AND METHODS

### 2.1 Host and parasitoid rearing

An *H. halys* colony was established from individuals collected at the Beijing Botanical Garden, China (E116°11′57″; N40°03′18″), in March 2017. They were maintained in continuous rearing on a diet of organic beans (*Phaseolus vulgaris* L.) and corn (*Zea mays* L.) at 25 ± 1°C, 60 ± 5% RH and16 L: 8 D photoperiod in gauze cages (60 × 60 × 60 cm). Egg masses were collected daily and used for parasitoid rearing.

Colonies of the two Asian parasitoids *T. japonicus* and *T. cultratus* were obtained from parasitized *H. halys* egg masses collected from a peach orchard in Beijing, China (N40°02′06″; E116°12′41″). The two species were separately maintained in transparent acrylic rearing cages (25 × 25 × 25 cm) and fed 20% honey water under the same rearing conditions as described above. To maintain the laboratory colonies, parasitoids were provided with fresh, laboratory‐reared *H. halys* egg masses twice per week. Specimens of *T. japonicus* and *T. cultratus* were taxonomically identified by E. Talamas (Florida Department of Agriculture and Consumer Service, Gainesville, FL, USA).

### 2.2 Intrinsic (larval) competition

Assays were carried out in small plastic Petri dishes (5 cm diameter), and observations were made using a stereomicroscope, following the experimental design of Konopka et al. (2017). Randomly selected *T. japonicus* females (2–7 days old, mated, naïve in terms of host oviposition) were offered fresh (<24 h) *H. halys* egg masses (12 eggs/mass) and observed until all eggs were parasitized, indicated by the characteristic marking behaviour. *Trissolcus cultratus* females (2–7 days old, mated, naïve) were provided egg masses previously parasitized by *T. japonicus* 0, 24, 48, 96, and 120 h earlier (16 to 21 replicates per interval) (note that 0 h refers to an egg mass that was parasitized by the first species and then immediately offered to the second species) and observed until they parasitized the entire egg mass or until females left the egg mass for >10 min. This approach ensured that parasitoids were provided parasitized hosts at different stages of embryonic development (i.e. parasitoid egg [0 h], larvae [24–72 h], pre‐pupae [96 h] and pupae [120 h]; as per Adidharma & Ciptadi, 2012; Konopka et al., 2020; Volkoff & Colazza, 1992). As controls, fresh *H. halys* egg masses (12 eggs/mass) were offered to *T. cultratus* (*n* = 20) and *T. japonicus* (*n* = 20) females separately to estimate emergence without competition. The number of eggs attempted (drilled) and parasitized (marked) by each *T. cultratus* female was recorded. Egg masses were kept for three weeks, and all parasitoid adults obtained were identified to species based on morphological characteristics (Talamas et al., 2017). The same experiment was repeated the opposite manner, providing eggs first to *T. cultratus* followed by *T. japonicus* (17 to 28 replicates per interval).

### 2.3 Extrinsic (adult) competition

Individual, 4 days old females of *T. japonicus* (*n* = 27) and *T. cultratus* (*n* = 21) were provided individually with single *H. halys* egg masses of regular size (28 eggs) in small plastic Petri dishes (5 cm diameter). Females were observed under the stereomicroscope until they parasitized all eggs and initiated brood guarding behaviour. Single females of the second species were then added to each dish through a small resealable hole on the side. Interactions of competing females were filmed for 30 min with a portable digital microscope and analysed with Observer XT software (Noldus Information Technology B.V., The Netherlands). Replicates in which wasps showed no interest in the egg mass (no searching behaviour, no interaction with the competitor) were excluded from the analysis.

For the guarding wasp (1^st^ species) the following parameters were recorded: guarding the egg mass, patrolling on the leaf, chasing competing wasp, fighting (direct physical contact with competitor) and walking off the leaf. For the competitor (2^nd^ species) added to the petri dish containing a guarding wasp, seven behavioural categories were defined: walking off the leaf, walking on the leaf, drumming on the egg mass, ovipositing, marking parasitized eggs, chasing guarding wasp and fighting. For both wasp species the last two categories were considered as aggressive behaviour towards the competitor. When the competing wasp was able to complete oviposition (indicated by marking) on at least one of the guarded eggs, extrinsic competition was considered successful.

### 2.4 Duration of guarding behaviour

Females of each parasitoid species (*T. japonicus*: *n* = 20; *T. cultratus*: *n* = 23) were exposed individually to fresh *H. halys* egg masses (28 eggs) in small Petri dishes (5 cm diameter) with a 5 mm hole in the lid, which was closed with a piece of foam. Females were observed until they had parasitized all eggs and started to guard the parasitized egg mass. Once guarding was observed, each plastic dish containing a wasp was placed individually in a larger 1.2‐L plastic container, the foam stopper on the petri dish was removed, and the larger container was sealed to prevent escape. Wasps were monitored every hour to determine whether they had left the egg mass and had entered the larger container, indicating that they were no longer guarding the egg mass.

### 2.5 Statistical analysis

The outcome of intrinsic and extrinsic competition was analyzed using R 4.0.5 (R Core Team, 2021). Simple generalized linear models for binomial distribution with logistic link function were built for each comparison of the intrinsic (larval) competition, followed by a postdoc test (Tukey's HSD of the ANOVA of the models) for the multiple comparisons of the predictors with controls. The extrinsic (adult) competition was analyzed using the parametric Pearson's Chi‐squared test, comparing the mean number of eggs laid by each species. The non‐parametric Kruskal‐Wallis rank sum test was used for the comparison of the number of eggs laid by ‘successful’ (=oviposition completed) wasps of each species as well as for the comparisons of different behaviours of the two species. For graphical presentation of results we used ggplot2.

## 3 RESULTS

### 3.1 Intrinsic (larval) competition

**FIGURE 1:**
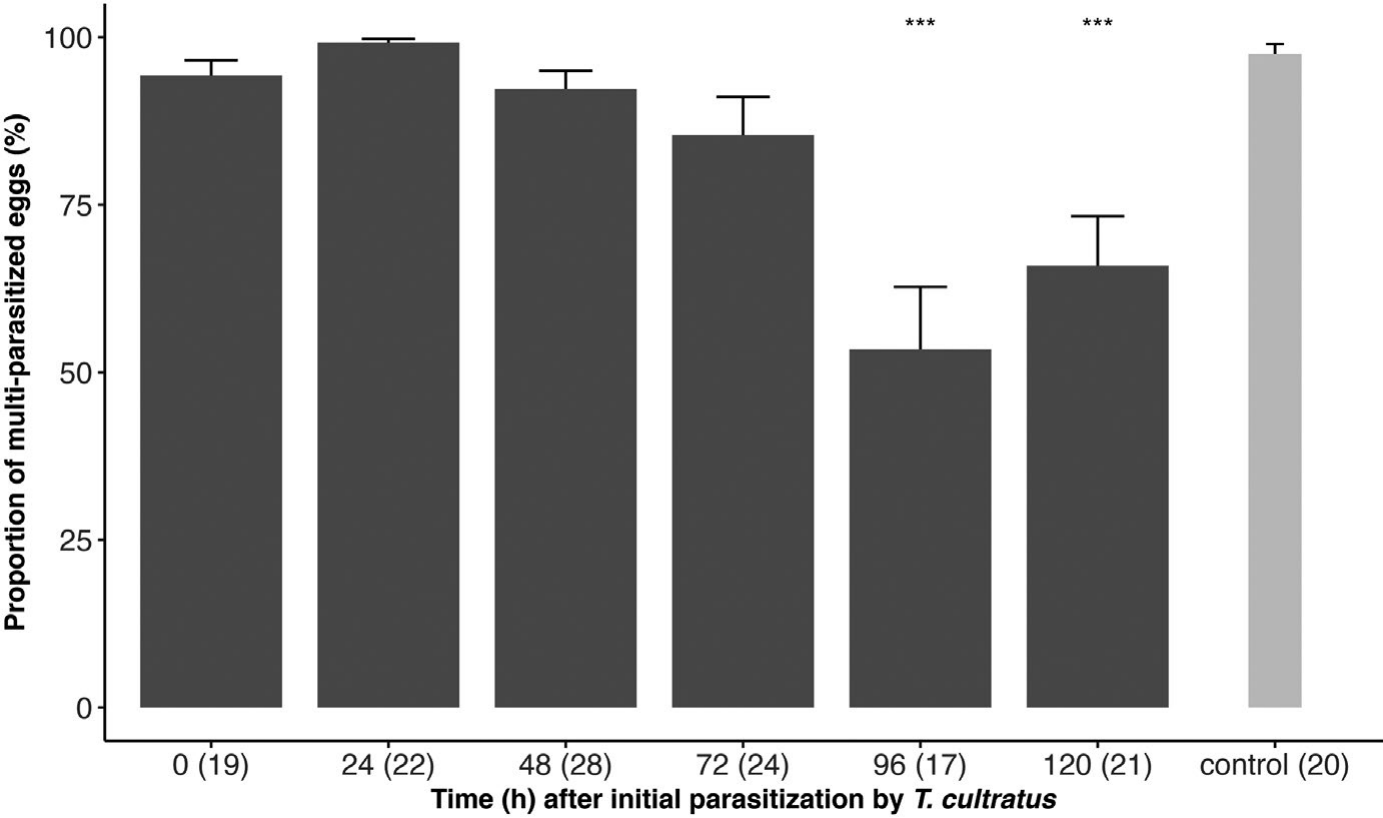
Mean proportion (±SE) of previously parasitized *Halyomorpha halys* egg masses attacked by *Trissolcus japonicus* (in brackets no. of multiparasitized egg masses). The asterisks indicate the level of significance (***<.001, **<.01, *<.05) of the comparison of the mean at each time interval to the control

**FIGURE 2:**
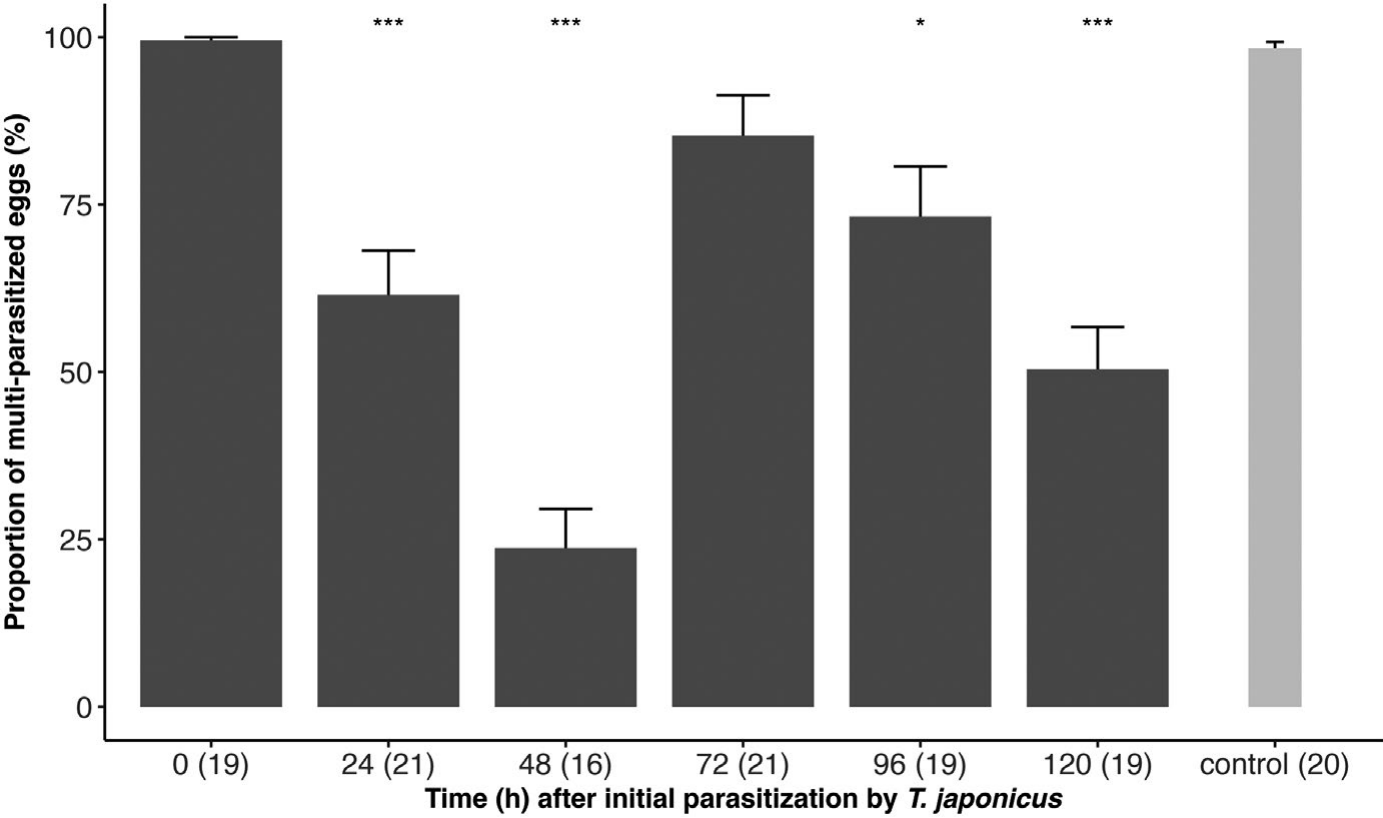
Mean proportion (±SE) of previously parasitized *Halyomorpha halys* egg masses attacked by *Trissolcus cultratus* (in brackets no. of multiparasitized egg masses). The asterisks indicate the level of significance (***<.001, **<.01, *<.05) of the comparison of the mean at each time interval to the control

*Trissolcus japonicus* females frequently attacked and marked *H. halys* egg masses that had been attacked by *T. cultratus* 0–72 h before. Acceptance levels ranged from 85% to 99%. However, after 96 h and 120 h the acceptance of previously parasitized eggs was significantly lower than in controls and dropped to 53% and 65%, respectively (Figure 1; Tukey's HSD Test for multiple comparisons on GLM: after 96 h: *p*‐adjusted < .001, 95% C.I. = [−0.66, −0.22]; after 120 h: *p*‐adjusted < .001, 95% C.I. = [−0.53, −0.11], Table S1). When egg masses were exposed to *T. cultratus* females immediately after they had been parasitized by *T. japonicus*, acceptance levels (99%) were not different from controls (Figure 2). However, if time delay was 24 h and 48 h, acceptance significantly dropped to 61% and 23%, respectively (Tukey's HSD test for multiple comparisons on GLM: after 24 h: *p*‐adjusted < .001, 95% C.I. = [−0.60, −0.14]; after 48 h: *p*‐adjusted < 0.001, 95% C.I. = [−0.98, −0.51], Table S2). In contrast, after 72 h acceptance increased again to 85% (Tukey's HSD test for multiple comparisons on GLM: after 72 h: *p*‐adjusted = .621, 95% C.I. = [−0.36, 0.10], Table S2), before it declined to 73% and 50% after 96 h and 120 h, respectively (Figure 2).

**FIGURE 3:**
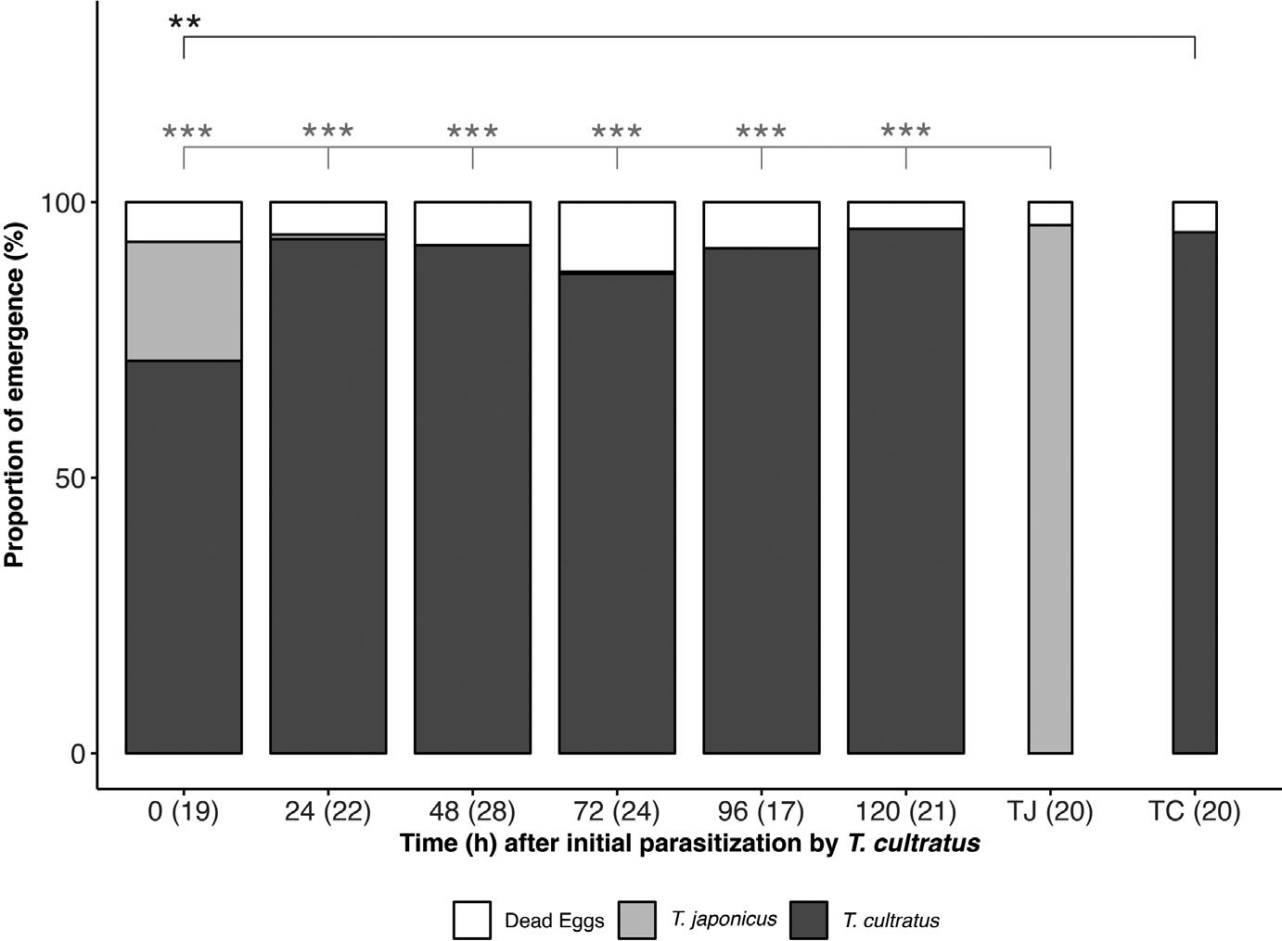
Mean proportion of *Halyomorpha halys* eggs resulting in no emergence, or emergence of *Trissolcus japonicus* or *Trissolcus cultratus*, following multiparasitism by *Trissolcus cultratus* (1^st^) and *Trissolcus japonicus* (2^nd^) at different time intervals (ages) between attacks. Sample sizes (= number of multiparasitized egg masses) are given in brackets. (TJ, TC: *Trissolcus japonicus* and *Trissolcus cultratus* rearing controls). The asterisks indicate the level of significance (***<.001, **<.01, *<.05) of the comparison of the mean for each time interval with controls

When eggs were first exposed to *T. cultratus* females and then immediately (0 h) to *T. japonicus*, 21% of multiparasitized eggs produced *T. japonicus* offspring (Figure 3, for statistics see Table S3). If the time delay was 24 h or more, this proportion decreased to <1% and the proportion of emerging *T. cultratus* was not different from controls.

**FIGURE 4:**
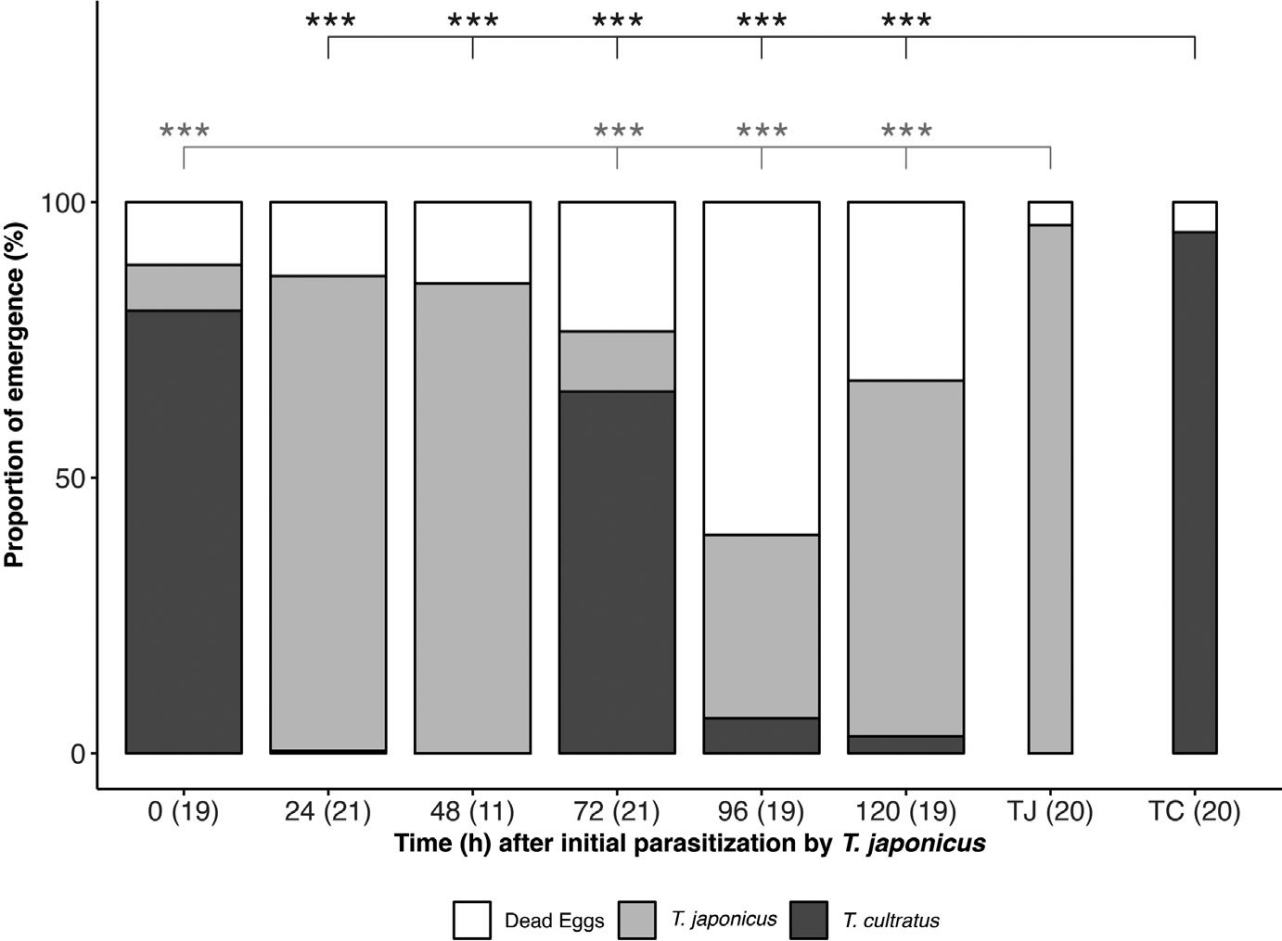
Mean proportion of *Halyomorpha halys* eggs resulting in no emergence, or emergence of *Trissolcus japonicus* or *Trissolcus cultratus*, following multiparasitism by *Trissolcus japonicus* (1^st^) and *Trissolcus cultratus* (2^nd^) at different time intervals (ages) between attacks. Sample sizes (= number of multiparasitized egg masses) are given in brackets. (TJ, TC: *T. japonicus* and *T. cultratus* rearing controls). The asterisks indicate the level of significance (***<.001, **<.01, *<.05) of the comparison of the mean for each time interval with controls

When eggs were exposed to the two parasitoids species in the reverse order (*T. japonicus* 1^st^), eggs which were multiparasitized by both species successively (0 h) produced primarily *T. cultratus* offspring. However, after 24 h and 48 h nearly all offspring were *T. japonicus*, and emergence was not different from *T. japonicus* controls (Tukey's HSD test for multiple comparisons on GLM: after 24 h: *p*‐adjusted = .877, 95% C.I. = [−0.12, 0.32]; after 48 h: *p*‐adjusted = .920, 95% C.I. = [−0.16, 0.37], Table S4). After a delay of 72 h, the proportion of *T. cultratus* offspring increased to 65%, whereas after 96 h and 120 h offspring emergence was dominated by *T. japonicus* but was nonetheless significantly lower than controls (Figure 4; Tukey's HSD test for multiple comparisons on GLM: after 72 h: *p*‐adjusted < .001, 95% C.I. = [0.63, 1.07]; after 96 h: *p*‐adjusted < .001, 95% C.I. = [0.40, 0.85]; after 120 h: *p*‐adjusted < .001, 95% C.I. = [0.088, 0.54], Table S4).

### 3.2 Extrinsic (adult) competition

The proportion of *T. japonicus* successfully ovipositing into eggs guarded by *T. cultratus* was 33.3% (*n* = 21), whereas 18.5% (*n* = 27) of *T. cultratus* oviposited into eggs guarded by *T. japonicus*; however, the observed differences were not significantly different (χ^2^ (1, *N* = 48) = 1.38, *p* = .2396). Successful *T. japonicus* females parasitized more eggs during the 30 min interval than *T. cultratus* (*T. japonicus*: 1–9 eggs per 30 min, mean = 3.86 ± 1.01, *n* = 7; *T. cultratus*: 1–4 eggs per 30 min; mean = 1.6 ± 0.6, n = 5; Kruskal‐Wallis rank sum test: H (1, *n* = 12) = 2.95, *df* = 1, *p* = .086).

**FIGURE 5:**
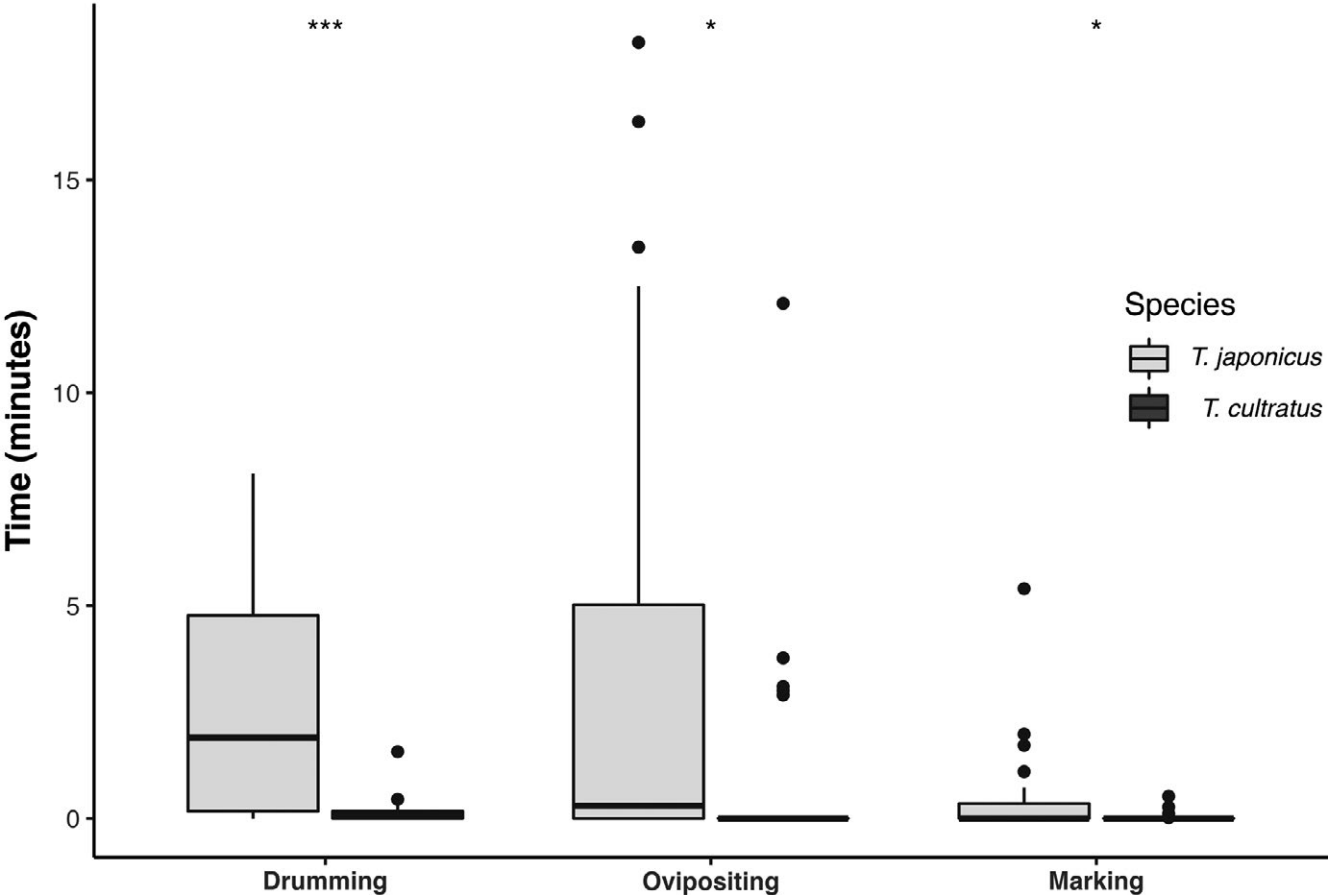
Time spent on drumming, ovipositing and marking on (guarded) *H. halys* egg masses by the competing wasps (2^nd^). TJ, *Trissolcus japonicus*; TC, *T*. *cultratus*

**FIGURE 6:**
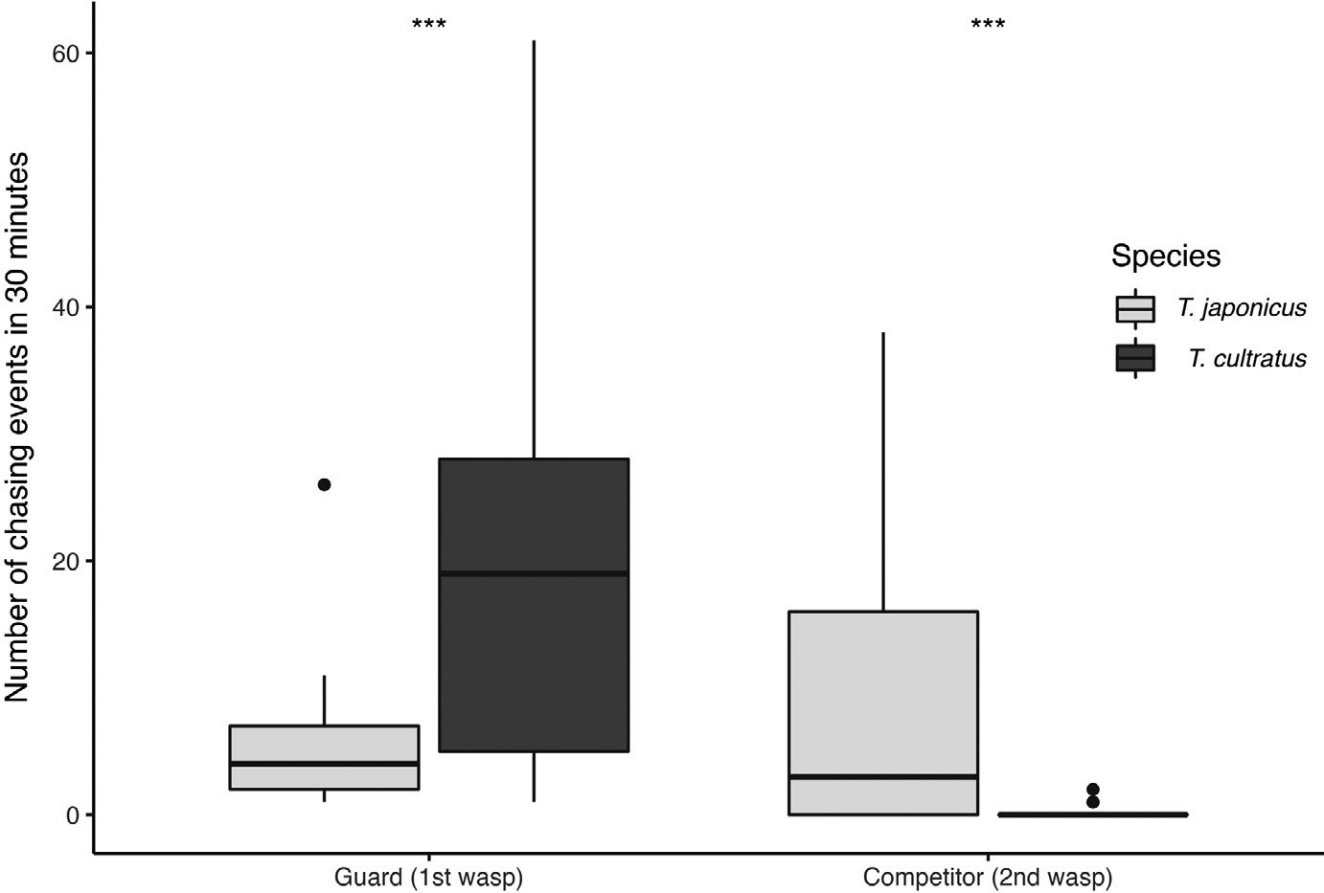
Number of chasing events for *Trissolcus japonicus* and *T. cultratus* when either guarding (1^st^) or competing (2^nd^) for the egg mass. TJ, *Trissolcus japonicus*; TC, *T*. *cultratus*

The two species also showed differences in their behaviour when competing against each other. Figure 5 shows the time spent on the *H. halys* egg mass by the second wasp when competing against the guarding wasp. *Trissolcus japonicus* spent significantly more time drumming (Kruskal‐Wallis rank sum test: H (1, *N* = 48) = 11.87, *df* = 1, *p* < .001) and ovipositing (Kruskal‐Wallis rank sum test: H (1, *N* = 48) = 8.43, *df* = 1, *p* = .004) than *T. cultratus*, but overall both species were rarely able to complete oviposition (Kruskal‐Wallis rank sum test: H (1, *N* = 48) = 2.38, *df* = 1, *p*‐value = .123). The two parasitoids showed significant differences in their aggressive behaviour when guarding or competing for the egg mass (Figure 6). When *T. japonicus* was guarding an egg mass, it chased *T. cultratus* off the egg mass less frequently than vice versa (Kruskal‐Wallis rank sum test: H (1, *N* = 27) = 11.1, *df* = 1, *p*‐value < .001). In contrast, when *T. japonicus* was the competing wasp it chased off the guarding wasp (*T. cultratus*) more frequently than vice versa (Kruskal‐Wallis rank sum test: H (1, *N* = 21) = 14.69, *df* = 1, *p*‐value < .001).

### 3.3 Guarding behaviour

**FIGURE 7:**
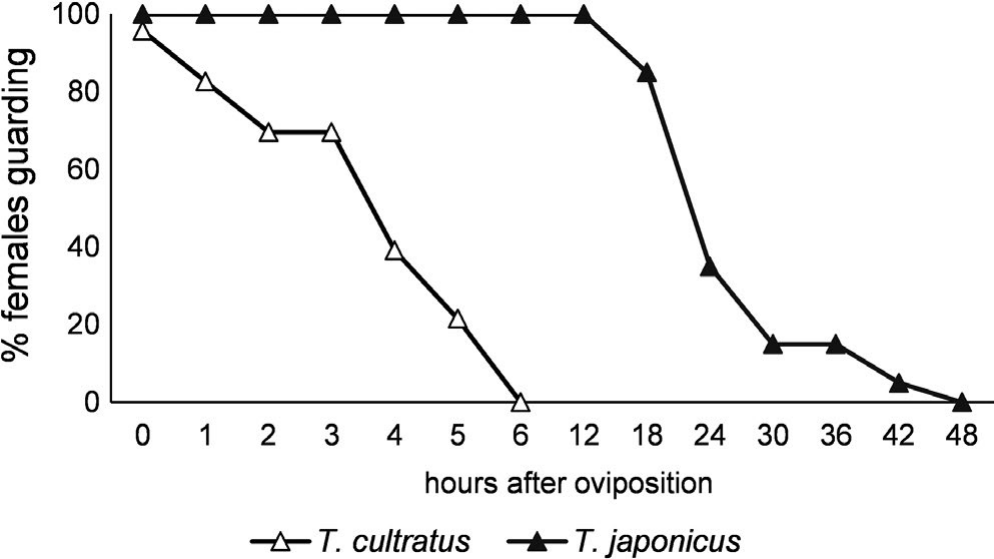
Proportion of *Trissolcus japonicus* (*n* = 20) and *T*. *cultratus* (*n* = 23) females guarding parasitized egg masses over a period of 48 h

*Trissolcus cultratus* females guarded egg masses for a maximum of 5 h, whereas the guarding period for *T. japonicus* females lasted a minimum of 12 h, and in some cases (~5%) >42 h (Figure 7).

## 4 DISCUSSION

### 4.1 Host acceptance

A high level of acceptance of previously parasitized eggs suggests that *T. japonicus* recognized the host as suitable, regardless of the presence of *T. cultratus* eggs and early‐stage larvae. Van Alphen and Visser (1990) suggest that external marking pheromones may not be recognized interspecifically. However, later stages of parasitoid development within the host should be detected by the ovipositing female – either due to the presence of the parasitoid, or to physiological changes that occur within the host (Hubbard et al., 1987). This may explain why *T. japonicus* accepted previously parasitized hosts in the earlier stages, as perhaps detection of the unsuitability was only possible in the later stages of parasitoid development when the parasitoid reached the pre‐pupal and pupal stages, and minimal host tissue remained in the egg (e.g. 72–120 h; Adidharma & Ciptadi, 2012; Konopka et al., 2020; Volkoff & Colazza, 1992).

When the order was reversed (*T. cultratus* exposed to host eggs previously parasitized by *T. japonicus*), high acceptance levels were only observed at 0 h and decreased significantly at the 24 and 48 h intervals. At the 24–48 h stage, the egg of the first species to parasitize has hatched into a mobile teleaform first instar larva with strong mandibles, which eliminates eggs and smaller larvae of competitors through physical combat (Volkoff & Colazza, 1992). The fact that *T. cultratus* avoids ovipositing in parasitized hosts indicates that *T. cultratus* perceives the parasitized host as less suitable for offspring development and survival and is therefore less likely to oviposit. Interestingly, acceptance of the parasitized host by *T. cultratus* changed again at 72 h and increased to >80%, indicating that parasitized hosts were again perceived as equally suitable as non‐parasitized hosts. At the 72 h interval the developing *T. japonicus* is in the third instar (hymenopteriform larva) and at the 96–120 h mark, the parasitoid is in the prepupal to pupal stages – which do not have mandibles and do not engage in physical combat with competitors (Konopka et al., 2017). At these stages host tissue has been consumed by the developing parasitoid (Konopka et al., 2017). This indicates a temporal trophic shift (Harvey et al., 2011) wherein *T. cultratus* switches from being a competitor to being an exploiter (hyperparasitoid) and accepts a host consisting of primary parasitoid tissue, rather than host tissue.

### 4.2 Developmental suitability

Regardless of the order, when both species are present as eggs (0 h interval), *T. cultratus* is the superior competitor, as indicated by successful development and emergence from multiparasitized eggs. This is presumably through direct physical combat between larvae upon hatching (Vinson, 1972), or due to differences in embryological period that result in one species hatching earlier and subsequently outcompeting the other species (Harvey et al., 2013). A shorter incubation time may give *T. cultratus* an advantage; this was the likely mechanism resulting in the competitive superiority of *Telenomus gifuensis* Ashmead (Hymenoptera: Scelionidae) over *Trissolcus nigripedius* Nakagawa (Mahmoud & Lim, 2008). The developmental biology of *T. japonicus* and *T. cultratus* have yet to be investigated, but studies pinpointing differences in embryology may shed some light on whether this contributes to the competitive superiority of *T. cultratus*. When *T. cultratus* parasitized the eggs first, all other intervals (24–120 h) yielded *T. cultratus* in proportions similar to those in control egg masses which were exposed solely to *T. cultratus*. This suggests that *T. cultratus* has a strong competitive advantage when it is the first species to parasitize. The ‘early–acting competitive superiority’ hypothesis, known in many host‐parasitoid systems (Harvey et al., 2013), occurs when the first parasitoid to oviposit has the developmental advantage and outcompetes the second parasitoid (van Baaren et al., 1995; Harvey et al., 2013). Elimination of the competitor could be through direct physical combat with the *T. cultratus* first instar larvae in the earlier intervals, or through physiological suppression (e.g. oxygen deprivation; starvation; secretion of toxic substances; Fisher, 1961) by sacciform second instar or hymenopteriform third instar *T. cultratus* (Volkoff & Colazza, 1992).

In the opposite scenario (*T. japonicus* first, followed by *T. cultratus*), lower acceptance by *T. cultratus* at 24 and 48 h intervals following parasitism by *T. japonicus* was accompanied by higher emergence of *T. japonicus* in multiparasitized eggs, suggesting that *T. japonicus* has the competitive advantage when it has the opportunity to develop to first instar larval stage prior to multiparasitism by *T. cultratus*. Furthermore, it suggests that *T. cultratus* is more selective in terms of oviposition in a parasitized host whereas *T. japonicus* does not discriminate between parasitized and unparasitized hosts for the first 72 h. The ability of *T. cultratus* to discriminate between parasitized and unparasitized hosts reduces the amount of resources invested in an unprofitable host and allows the parasitoid to disperse and forage on more profitable (unparasitized) host patches (van Lenteren, 1990). Multiparasitism and superparasitism are only adaptive when unparasitized hosts are scarce and when survival of the second species in an already‐parasitized host is highly likely (Hubbard et al., 1987). Although this suggests that the behaviour of *T. japonicus* may be maladaptive, as the parasitoid female wastes her eggs in a host that is unsuitable for development, it is possible that this behaviour may be overestimated under laboratory conditions. For example, Sujii et al. (2002) showed that although *T. basalis* (Wollaston) will readily multiparasitize under laboratory conditions, this may be overestimated due to oviposition pressure. Further, Laumann et al. (2008) have shown that species of *Trissolcus* differ in their ability to exploit a shared host resource based on egg load, and a higher fecundity was observed for competitively superior *T. basalis* in comparison to *T. brochymenae* (Ashmead), *T. teretis* Johnson, and *T. urichi* (Crawford). A better understanding of fecundity and egg load, and how they relate to oviposition pressure in *T. japonicus* and *T. cultratus* may help to put the observed behaviours into context.

The outcome of intrinsic competition reaffirms the vulnerability of third instar *T. japonicus* (72 h), as *T. cultratus* successfully emerges from multiparasitized eggs, confirming the adaptive outcome of the temporal trophic shift from primary to facultative hyperparasitoid. The success of this trophic shift ends following pupation of *T. japonicus*, with a detrimental outcome for both species, as non‐reproductive mortality increases significantly (~60% at 96 h). This is consistent with Agboka et al. (2002) who observed high levels of host and parasitoid mortality with later‐stage multiparasitism by species of *Telenomus* Haliday. Facultative hyperparasitism as a competitive strategy has been observed when European *T. cultratus* competes with Asian *T. japonicus*, despite the fact that they have not co‐evolved as competitors (Konopka et al., 2017). Although European populations of *T. cultratus* have not retained the ability to develop in *H. halys* eggs (Talamas et al., 2019), the existence of a temporal trophic shift in both Asian and European populations suggests that facultative hyperparasitism as a competitive strategy has been conserved across geographically‐diverse populations of *T. cultratus*.

The ability of an egg parasitoid to develop as a facultative hyperparasitoid has not been shown in other *Trissolcus* species. However, Viktorov (1966) reported the scelionid *Telenomus chloropus* Thomson (syn. *Te. sokolovi* Mayr) develops as a hyperparasitoid on *Trissolcus semistriatus* Nees (Johnson, 1984), and encyrtid (Hymenoptera) egg parasitoids of Pentatomidae display the ability to develop as facultative hyperparasitoids on scelionid primary parasitoids. Cusumano et al. (2011), Cusumano et al. (2013) demonstrated that not only is *Ooencyrtus telenomicida* (Vassiliev) (Hymenoptera: Encyrtidae) the superior intrinsic competitor in comparison to *T. basalis*, but it can also develop as a facultative hyperparasitoid on the latter species, increasing the window of opportunity to exploit host eggs and avoiding exclusion by the more fecund and superior extrinsic competitor *T. basalis*. Similarly, Mohammadpour et al. (2014) investigated competition between *Ooencyrtus pityocampi* (Mercet) (Hymenoptera: Encyrtidae) and *T. agriope* (Kozlov & Lê) on host eggs of *Brachynema signatum* Jakovlev (Hemiptera: Pentatomidae) and reported the development of *O. pityocampi* as a facultative hyperparasitoid on *T. agriope*, but not the other way around. Development as a facultative hyperparasitoid is often avoided due to the associated fitness costs, including reduced number of progeny, smaller females, and longer developmental times (Cusumano et al., 2013, 2015). As host tissues are being converted from the second to the fourth trophic level, these fitness costs are likely due reduced resources (Harvey et al., 2011), as this scenario represents an extreme example of resource sharing by proxy. As such, the occurrence of facultative hyperparasitism under field conditions is potentially rare and may be advantageous only when resources are scarce (van Alphen & Visser, 1990).

### 4.3 Guarding behaviour

Intrinsic competition can only take place if a parasitoid has access to a parasitized egg mass. Scelionid females typically guard an egg mass following parasitization to deter competitors; however, time spent guarding and defending an egg mass is at the expense of continued foraging and exploitation (Field, 1998). Therefore, the length of time spent guarding should be long enough to ensure a developmental advantage for their progeny, but not so long as to compromise subsequent foraging and reproductive output. In an extreme example, in the presence of *Trissolcus* spp. competitors, *Psix tunetanus* (Mineo & Szabo) (Hymenoptera: Scelionidae) aggressively guards a pentatomid egg mass until progeny emergence to secure the egg mass for their offspring, but severely compromise their reproductive output by doing so (Jones & Sieglaff, 1991). It is more typical for species of *Trissolcus* (e.g. *T. basalis*, *T. utahensis* [Ashmead], *T. plautiae* [Watanabe], *T. semistriatus* [syn. *T. nigripedius*]) to guard an egg mass for <24 h (Jones & Sieglaff, 1991; Mahmoud & Lim, 2008; Ohno, 1987). Guarding behaviour is important in mediating further in‐host competitive interactions. For example, *T. semistriatus* will guard an egg mass up to 11 h in the presence of the superior intrinsic competitor *Te. gifuensis*, and the outcome of this guarding is a higher proportion of successfully developed *T. semistriatus*, thereby making it the superior extrinsic competitor (Mahmoud & Lim, 2008). The two *Trissolcus* species observed in the present study differ markedly in the length of time they spend guarding the egg mass. *Trissolcus cultratus* spent 1–5 h guarding an egg mass, whereas *T. japonicus* guarded for 12–42 h. This is congruent with the outcome of intrinsic competition when *T. cultratus* was the first species to parasitize, as this species was competitively superior in all post‐parasitism intervals, indicating that prolonged patch guarding would not be an efficient strategy to maximize lifetime reproductive success. In contrast, *T. japonicus* guarded parasitized egg masses 8–12× longer than *T. cultratus* and remained on the egg mass long enough to ensure a developmental advantage in the event of multiparasitism. However, the duration of guarding by *T. japonicus* does not prevent hyperparasitism by *T. cultratus* around the 72 h mark, as presumably the trade‐off between ensuring the survival of a single cohort of offspring versus exploitation of fresh egg masses becomes too high (Field et al., 1998; Vet et al., 2002).

Despite the fact that *T. japonicus* females are known to be aggressive competitors (Konopka et al., 2017), the present study showed that guarding *T. japonicus* were actually less aggressive towards competing *T. cultratus* than vice versa. This may be explained by the slow ‘stalking’ movement of *T. cultratus* when it encounters a guarded egg mass, which may attract less attention by the guarding wasp. In contrast, *T. japonicus* tends to ‘ambush’ the guarded egg mass and moves fast and directly towards the guarding wasp, which resulted in more frequent chasing events by *T. cultratus* to prevent *T. japonicus* from accessing the egg mass. The results of intrinsic competition suggest that aggressive behaviour of *T. japonicus* towards guarding *T. cultratus* could benefit *T. japonicus* if eggs were only recently parasitized. Approximately 20% of multiparasitized eggs that were parasitized by *T. cultratus* followed by *T. japonicus* yielded *T. japonicus* offspring, but only when the two attacks (parasitism indicated by marking behaviour following oviposition) took place fairly close together (i.e. within hours of each other). Although the odds are not in favour of *T. japonicus*, depending on the physiological state of the female, it may be less risky than leaving the patch in search of an unparasitized egg mass (Goubault et al., 2004, 2005). This is consistent with the ‘desperado’ effect, wherein the individual risks being without resources unless they initiate an aggressive encounter (regardless of outcome), particularly if resources are scarce and of relatively high value (Grafen, 1987; Morrell et al., 2005).

Neither species were particularly successful in completing oviposition on guarded eggs, as chasing events by the guarding species were highly effective at preventing access of the intruder. This has also been shown in pairwise contests with other species of *Trissolcus* (e.g. *T. utahensis*, *T. basalis*) where the first species guards and successfully defends the resource in the presence conspecifics, also referred to as the ‘bourgeois’ rule (Field & Calbert, 1999; Field et al., 1998; Hardy et al., 2013; Jones & Sieglaff, 1991).

## 5 CONCLUSIONS AND FUTURE DIRECTIONS

Collectively, the data generated in these experiments demonstrated that *T. cultratus* is the superior intrinsic competitor when it is the first species to parasitize an egg mass as a primary parasitoid, and also when it has the opportunity to develop as a facultative hyperparasitoid. This species demonstrates a short guarding time and more aggressive chasing events when it is the first parasitoid to attack an egg mass. However, *T. cultratus* is less likely to engage in aggressive behaviour when it is the second species to arrive, suggesting that *T. cultratus* invests fewer resources in extrinsic competition with another female on an already‐parasitized egg mass. In contrast, *T. japonicus* is the superior extrinsic competitor, and adult females invest more resources in patch guarding, as evidenced by a longer guarding time following parasitism, and more aggressive attempts to oviposit in a guarded, parasitized egg mass. This is consistent with counterbalance competition (Zwoelfer, 1971), wherein coexistence is possible if an inferior larval competitor evolves superior host exploitation abilities as an adult. Interestingly, some of the behaviours displayed by *T. japonicus* appear to be maladaptive and may be inefficient as competitive strategies. For example, *T. japonicus* does not avoid oviposition in hosts already parasitized by *T. cultratus* (up to 72 h post‐parasitism), despite the fact that developmental success is virtually non‐existent. Furthermore, *T. japonicus* engages in more aggressive interactions and spends more time foraging on guarded (already‐parasitized) egg masses despite the fact that oviposition is rarely successful and developmental outcome is poor. However, this may be an artefact of increased oviposition pressure in no‐choice tests or small‐cage arenas (Murray et al., 2010; Rojas‐Rousse, 2010), or it may be due to a high egg load or low egg retention capacity in the absence of suitable hosts (Agboka et al., 2002; Collier et al., 2002; Stokkebo & Hardy, 2000). Choice tests in future studies may help clarify whether *T. japonicus* is able to discriminate between suitable (unparasitized) and unsuitable (parasitized) host eggs. Furthermore, a better understanding of differences in the fecundity and developmental biology of *T. japonicus* and *T. cultratus* may provide insight on the reproductive strategies displayed by these species when they compete for a common host resource. In the case of *T. cultratus*, investigating differences in developmental biology and fitness‐related proxies as a primary parasitoid versus facultative hyperparasitoid would be of value to determine the consequences of developing at the fourth trophic level and may provide insight as to how common this developmental strategy actually is in the field.

## CONFLICT OF INTEREST

The authors declare no conflict of interest.

## AUTHOR CONTRIBUTIONS

**Tim Haye:** Conceptualization (equal); Funding acquisition (equal); Investigation (equal); Methodology (equal); Project administration (equal); Resources (equal); Supervision (equal); Validation (lead); Visualization (equal); Writing – original draft (equal); Writing – review & editing (equal). **Jinping Zhang:** Conceptualization (supporting); Investigation (equal); Writing – review & editing (supporting). **Marion Risse:** Formal analysis (lead); Methodology (supporting); Visualization (equal); Writing – original draft (supporting). **Tara D. Gariepy:** Conceptualization (equal); Funding acquisition (equal); Investigation (supporting); Methodology (equal); Project administration (equal); Resources (equal); Supervision (equal); Writing – original draft (equal); Writing – review & editing (equal).

## Supporting information

Table S1–S4

## Data Availability

Data from experiments on parasitoid acceptance, emergence, and guarding behaviour have been deposited in Dryad (https://doi.org/10.5061/dryad.gmsbcc2p4).
