# Supplementary material for: A temporal trophic shift from primary parasitism to facultative hyperparasitism during interspecific competition between two coevolved scelionid egg parasitoids
Source: Ecol Evol. 2021 Dec 20;11(24):18708–18. doi: 10.1002/ece3.8483 (PMC8717335; doi:10.1002/ece3.8483)
Supplement: Supplementary file 1 — Table S1–S4 [file ECE3-11-18708-s001.docx]

**Supplementary Table 1** Tukey’s HSD Test for multiple comparisons on the generalized linear model of the number of multiparasitzed egg masses attacked by *T. japonicus* at different time points (predictors) after first parasitisation by *T. cultratus* compared to the number of egg masses parasitized by *T. japonicus* without prior parasitisation by the other species (control).

| Predictor | Diff | 95 % CI | p-value |
| --- | --- | --- | --- |
| 0h | -0.0320 | [-0.2474, 0.1834] | 0.9994 |
| 24h | 0.0174 | [-0.1903, 0.2252] | 0.9999 |
| 48h | -0.0524 | [-0.2492, 0.1445] | 0.9851 |
| 72h | -0.1208 | [-0.3244, 0.0828] | 0.5671 |
| 96h | -0.4407 | [-0.6625, -0.2189] | 0.0000 |
| 120h | -0.3163 | [-0.5264, -0.1062] | 0.0003 |

**Supplementary Table 2** Tukey’s HSD Test for multiple comparisons on the generalized linear model of the number of multiparasitzed egg masses attacked by *T. cultratus* at different time points (predictors) after first parasitisation by *T. japonicus* compared to the number of egg masses parasitized by *T. cultratus* without prior parasitisation by the other species (control).

| Predictor | Diff | 95 % CI | p-value |
| --- | --- | --- | --- |
| 0h | 0.0123 | [-0.2236, 0.2482] | 0.9999 |
| 24h | -0.3683 | [-0.5983, -0.1382] | 0.0001 |
| 48h | -0.7458 | [-0.9787, -0.5130] | 0.0000 |
| 72h | -0.1302 | [-0.3602, 0.0999] | 0.6213 |
| 96h | -0.2509 | [-0.4868, -0.0150] | 0.0292 |
| 120h | -0.4789 | [-0.7149, -0.2430] | 0.0000 |

**Supplementary Table 3** Tukey’s HSD Test for multiple comparisons on the generalized linear models of the number of wasps of each species emerging from the multiparasitzed egg masses by *T. japonicus* at different time points (predictors) after first parasitisation by *T cultratus* compared to the number of wasps emerging from egg masses parasitized by one single species (controls).

| Predictor | Control *T. japonicus* | | | Control *T. cultratus* | | |
| --- | --- | --- | --- | --- | --- | --- |
|  | Diff | 95 % CI | p-value | Diff | 95 % CI | p-value |
| 0h | 7.4262e-01 | [0.6363, 0.8490] | 0.0000 | 0.2207 | [0.0491, 0.3922] | 0.0029 |
| 24h | 9.5041e-01 | [0.8478, 1.0530] | 0.0000 | 0.0001 | [-0.1654, 0.1656] | 1.0000 |
| 48h | 9.5833e-01 | [0.8611, 1.0555] | 0.0000 | 0.0079 | [-0.1489, 0.1647] | 0.9999 |
| 72h | 9.5486e-01 | [0.8543, 1.0554] | 0.0000 | 0.0597 | [-0.1024, 0.2219] | 0.9490 |
| 96h | 9.5833e-01 | [0.8488, 1.0679] | 0.0000 | 0.0167 | [-0.1600, 0.1934] | 0.9999 |
| 120h | 9.5833e-01 | [0.8546, 1.0621] | 0.0000 | -0.0183 | [-0.1857, 0.1490] | 0.9999 |

**Supplementary Table 4** Tukey’s HSD Test for multiple comparisons on the generalized linear models of the number of wasps of each species emerging from the multiparasitzed egg masses by *T. cultratus* at different time points (predictors) after first parasitisation by *T. japonicus* compared to the number of wasps emerging from egg masses parasitized by one single species (controls).

| Predictor | Control *T. japonicus* | | | | Control *T. cultratus* | | |
| --- | --- | --- | --- | --- | --- | --- | --- |
|  | Diff | 95 % CI | p-value | Diff | | 95 % CI | p-value |
| 0h | 0.8750 | [0.6495, 1.1005] | 0.0000 | 1.3032e-01 | | [-0.0122, 0.2728] | 0.0998 |
| 24h | 0.0967 | [-0.1232, 0.3166] | 0.8765 | 9.2863e-01 | | [0.7896, 1.0676] | 0.0000 |
| 48h | 0.1061 | [-0.1581, 0.3702] | 0.9200 | 9.3295e-01 | | [0.7660, 1.0999] | 0.0000 |
| 72h | 0.8494 | [0.6269, 1.0720] | 0.0000 | 2.7668e-01 | | [0.1360, 0.4173] | 0.0000 |
| 96h | 0.6258 | [0.4004, 0.8513] | 0.0000 | 8.6906e-01 | | [0.7265, 1.0116] | 0.0000 |
| 120h | 0.3129 | [0.0874, 0.5384] | 0.0009 | 9.0225e-01 | | [0.7597, 1.0448] | 0.0000 |
